# A dataset of 40’000 trees with section-wise measured stem diameter and branch volume from across Switzerland

**DOI:** 10.1038/s41597-024-03336-7

**Published:** 2024-05-09

**Authors:** Markus Didion, Anne Herold, Esther Thürig, Serra Topuz, Zeljka Vulovic, Meinrad Abegg, Jens Nitzsche, Jonas Stillhard, Jonas Glatthorn

**Affiliations:** Forest Resources and Management, Swiss Federal Institute for Forest, Snow and Landscape Research WSL, Birmensdorf, Switzerland

**Keywords:** Forest ecology, Forestry

## Abstract

Estimating growing stock is one of the main objectives of forest inventories. It refers to the stem volume of individual trees which is typically derived by models as it cannot be easily measured directly. These models are thus based on measurable tree dimensions and their parameterization depends on the available empirical data. Historically, such data were collected by measurements of tree stem sizes, which is very time- and cost-intensive. Here, we present an exceptionally large dataset with section-wise stem measurements on 40’349 felled individual trees collected on plots of the Experimental Forest Management project. It is a revised and expanded version of previously unpublished data and contains the empirically derived coarse (diameter ≥7 cm) and fine branch volume of 27’297 and 18’980, respectively, individual trees. The data were collected between 1888 and 1974 across Switzerland covering a large topographic gradient and a diverse species range and can thus support estimations and verification of volume functions also outside Switzerland including the derivation of whole tree volume in a consistent manner.

## Background & Summary

Forests are an important global resource and information on forests has been collected in inventory systems for decades or centuries following country-specific approaches^1^. The growing stock comprising the volume of stems of standing trees over a specific forest area^2^, is, recognised as one of the most important variables in forest inventories, particularly in Europe^3,4^. Growing stock serves as an indicator of forest functions^4^, as the basis for the development of forest management practices^5^, policy making^6^, and international reporting^7^. Data on individual stems is used to study, for example, tree taper^8^, volume^9^, and growth^10^.

Growing stock and in particular the volume of individual trees cannot be measured directly. Tree volume is thus usually estimated using models developed on the basis of tree attributes that can be measured in the field. These typically include diameter at breast height (DBH), and in some countries a diameter at a second height, and total tree height^4,11^. Volume models are, however, generally developed based primarily on local data that are not representative on a national scale and of the occurring tree species^12^. A reason for this is that representative large-scale sampling is typically too time-consuming and costly^11^. While methods for estimating the volume of stems have been developed accounting for these limitations, this is much less the case for branch volume and crown mass. Since growing stock in European forest inventories excludes the stump and branches^2^, it underestimates total above-ground tree volume. To also account for branches additional measurements are needed. Branch volume is typically estimated using separate functions or expansion factors^4^. These are generally derived from independent data based on different and typically limited population samples^13^. Making existing datasets available to the scientific community has the potential to significantly contribute to further develop existing methods.

The Swiss National forest inventory (NFI) is the main source of nationally representative information on the state and change of forest volume, biomass, and carbon stocks in Switzerland. Data from the NFI are the basis for several research, monitoring, and reporting programs such as national and international forest reports and greenhouse gas reporting. In addition to the classic assessment of growing stock, accurate estimates of whole-tree biomass and carbon stocks are therefore required. The methods applied in the Swiss NFI are continuously improved and regularly documented e.g., Brassel and Lischke^14^, Fischer and Traub^15^. The volume of above-ground coarse (i.e. ≥7 cm in diameter, including tree stump) and fine woody parts of stem and branches is estimated using functions fitted to data collected on sites of the Experimental Forest Management project’s (EFM) long-term growth and yield plot network^16^. The EFM project collects growth and yield data in Switzerland since the late 1880’s on more than 1’000 plots^17,18^. In addition to monitoring data of standing living trees, detailed measurements of felled trees were conducted in the past. The EFM is an ongoing project and long-term consistency is assured.

This data paper presents an exceptionally large dataset with measurements of individual trees combining stem size (diameter and length) with the volume of coarse (converted from measured size) and fine (converted from measured weight) branches. This dataset differs from the one used to derive volume functions for stem- and branchwood in the Swiss NFI^16,19^ in that the previously separate stem- and branchwood datasets are linked at the level of individual trees. The dataset also includes additional tree measurements and metadata. By linking stem- and branchwood measurements for individual trees, a consistent total above-ground tree volume can be derived. This information can be used to evaluate the accuracy of typical approaches to obtain total tree volume, such as adding up estimates based on two separate models or applying expansion factors which are based on different tree populations. The data can be used to further develop existing volume estimates in the Swiss NFI, resulting in higher accuracy of derived variables such as biomass and C stocks. Open access to the dataset can also support the estimation and verification of volume functions also outside Switzerland, as growing stock and total tree biomass are among the most important variables in forest inventories^4^.

## Methods

Starting in the 1880s, the EFM project is one of the longest running scientific projects in Switzerland with the primary objective to provide long‐term empirical data to examine forest development under the influence of management and changing environmental conditions^17^. Besides repeated measurements on living trees, comprehensive measurements on felled trees have also been conducted throughout the years, which are the object of this data paper. Individual tree data for a range of tree species (Table 1) were obtained following the field procedure described in Flury^20,21^. First, all trees were numbered and the stem was marked at the height of 1.30 m measured from ground-level. At the height of 1.30 m the DBH was measured crosswise in millimetres using callipers with the first diameter horizontally and the second in the direction of the slope; on a slope the measurement was on the uphill side^22,23^. Trees to be felled were selected based on Urich’s method^24,25^ which uses DBH classes for obtaining a representative selection. On a subset of the felled trees, detailed length and diameter measurements on coarse stem and branch parts as well as weights of fine parts were obtained (see section ‘Data records’ and Table 2).

**Table 1:** Tree species information.

Species ID	Species name	NFI main species	N
21	*Picea abies*	Picea	15’684
22	*Abies alba*	Abies	7’344
23	*Pinus sylvestris*	Pinus spp	1’657
24	*Larix decidua*	Larix	1’629
25	*Pinus strobus*	Pinus spp	847
26	*Pseudotsuga menziesii*	other conifers	601
27	*Pinus cembra*	P. cembra	224
28	*Pinus mugo* Turra subsp. mugo	Pinus spp	103
29	*Picea sitchensis*	Picea	29
30	*Pinus nigra*	Pinus spp	129
31	*Abies grandis*	Abies	61
32	*Chamaecyparis*	other conifers	60
33	*Cryptomeria japonica*	other conifers	21
34	*Thuja plicata*	other conifers	77
35	*Picea omorika*	Picea	14
36	*Larix kaempferi* (Lamb.) Carrière	Larix	4
41	*Fagus sylvatica*	Fagus	8’603
42	Quercus *petraea, Q. robur*, Q. *rubra*	Quercus	1’821
43	*Fraxinus americana*, F. *excelsior*	Fraxinus	153
44	*Acer campestre*, A. *platanoides*, A. *pseudoplatanus*	Acer	96
45	*Populus tremula*	other broadleaves	216
46	*Castanea sativa*	Castanea	82
47	*Betula pendula*	other broadleaves	97
48	*Juglans regia*	other broadleaves	218
51	*Ulmus glabra*	other broadleaves	8
52	*Prunus avium*	other broadleaves	4
60	Other broadleaves, incl. *Sorbus* spp and *Tilia* spp	other broadleaves	9

Species are grouped based on the classification used in the Swiss National Forest Inventory (Table 14.1 in Didion, *et al*.^37^). Species was not recorded for 558 trees.

**Table 2:** Tree specific (total N = 40’349) data observed or measured with units in brackets.

Variable name	Definition	Value range	N
TreeID	Running number	1–40’349	40’349
TreeSpecies	Species name	See Table 1	39’791
NFI_mainspecies	NFI main species	See Table 1	40’349
TreeAge	age [years]	1–43–65–96–340	33’143
DBH	Mean DBH [mm]	6–138–230–341–1581	40’349
H_total	Total height [dm]	15–151–226–284–574	40’349
L_coarsestem	Length of stem from the base to stem D = 7 cm [dm]^+^	0–106–192–252–552	40’305
L_coarsestemfinal	Length of the final section of the stem until D = 7 cm [dm] if not 2 m in length	0–0–6–10–186	40’305
L_top	Length of the tree top (part of the stem where D < 7 cm [dm]	2–26–36–46–293	40’305
DM065, DM1, DM3, … DM53	mean stem D at 0.65 m and every 2 m starting at 1 m where D ≥ 7 cm [mm]	Fig. 6	Fig. 6
D_coarsestemfinal	D of the final section of the stem until D = 7 cm [mm] measured at half its length	0–0–73–79–175	39’751
D_top	D of the tree top (part of the stem where D < 7 cm measured at half its length [mm]	0–32–37–42–96	39’751
V_coarsebranch	Volume of coarse branchwood ≥ 7 cm in diameter [dm3]	Fig. 7	27’297
V_finewoodytotal	Total volume of fine woody elements < 7 cm in diameter, i.e. including tree top [dm3]	Fig. 7	18’980
V_finebranch	Volume fine branchwood < 7 cm in diameter [dm3]	0–0–5–147–4210	9’667

For continuous tree data, the value range shows minimum, quartiles, and maximum. D indicates diameter.

**Fig. 1:**
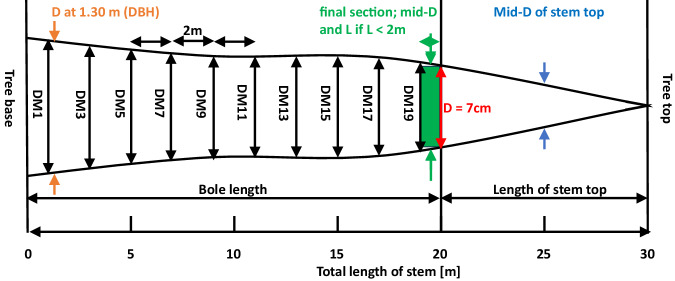
Measurements along the stem. Length below 7 cm diameter (D) from the base of the tree, i.e. including stump; section-wise diameter every 2 m along the stem starting at 1 m from the base of the tree: DM1, DM3, DM5, etc. Additional measurements: Diameter at 0.65 m and 1.30 m (DBH); mid-diameter and length of the final stem section where D ≥ 7 cm if the length is <2 m; and mid-diameter and length of stem top.

**Fig. 2:**
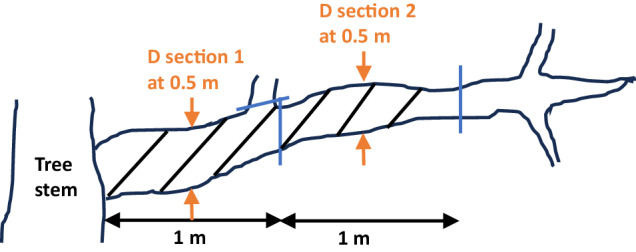
Measurements along the branch. Each branch and each side-branch was divided in 1 m long sections and the section diameter was recorded at 0.5 m. Branch sections with a diameter ≥7 cm were recorded as coarse branches (indicated by diagonal lines). Smaller pieces were removed (indicated with blue lines) and accounted as small branches measured separately in standardized bundles.

The coarse woody part of the stem starting from the base of a tree up to the diameter threshold of 7 cm (i.e. including stump and bole following Gschwantner, *et al*.^26^) was divided into sections of 2 m length and the diameter at half the length of each section was measured crosswise (Fig. 1). The section-wise diameter measurements therefore started at 1 m from the tree base and were continued along the stem up to the thinner end where the diameter was 7 cm. As the final section was considered where the coarse stemwood diameter reached the lower bound of 7 cm. If the length of the final section was <2 m, its full length and diameter at half its length were measured. On a subset of the trees an additional diameter measurement was made at 0.65 m. Section-wise measurements were also made on coarse branches but based on a section length of 1 m (Fig. 2). Decisive for the attribution to coarse branchwood was the diameter at half the length of a 1 m section, which was also used for deriving the cylindric volume^21^.

**Fig. 3:**
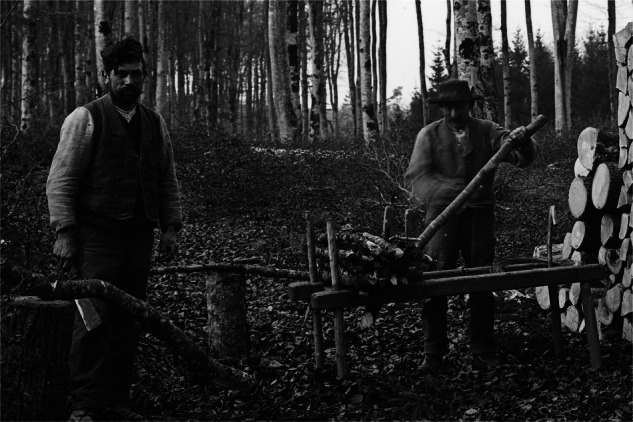
Photograph illustrating the production of standardized bundles of small (<7 cm in diameter) woody parts of stem, i.e tree top, and branches. Photo ‘*Wellenmacher an der Arbeit*’ (Preparation of bundles) in a Bernese forest by Herrmann Knuchel, 17.11.1916 from the collection of the Swiss Federal Research Institute WSL, reference no. EAF_00831_G_neg.

The parts of stem and branches below the diameter threshold of 7 cm (henceforth stem top and fine branches, respectively, including needles or leaves) were collected and fitted into standardised bundles of ca. 1 m length and ca. 1 m circumference (Fig. 3). The fresh weight of bundles was measured directly in the field. Conversion factors (Table 3) were used to calculate the volume of standardized bundles from their fresh weight^27^. These were derived from data collected in the years 1888 to 1892. The data comprised both, measured fresh weight and xylometric volume for a total of 2192 standardized bundles with fine woody material collected on a representative subset of the EFM sites for the tree species *Picea*, *Abies*, *Pinus*, *Fagus*, and *Fraxinus*. The conversion factors derived by Flury^27^ were reviewed in 1940 and expanded with more precise data for additional species. Revised factors (Table 3) were based on data from Gayer and Fabricius^28^. Since the factors after Gayer and Fabricius^28^ were developed for stemwood, values were slightly modified based on expert knowledge for the application to fine woody material. The revised factors were applied for weight to volume conversion starting 1940.

**Table 3:** Conversion factors based on Flury^27^ used until 1940 and E. Badoux (Forest engineer growth and yield, Federal Institute for Forest Research, predecessor of WSL) used after 1940 to calculate the volume of fine woody (i.e. diameter <7 cm) stem and branch material from field measurements of the fresh weight [kg] of collected standardized bundles of 1 m length and 1 m circumference [m^3^].

Tree species	Conversion Factor	
	Flury^27^	Badoux
*Picea* spp.	0.9	0.9
*Abies* spp.	0.9	0.9
*Pinus* spp.	0.9	0.9
*Larix* spp.	as *Picea*	0.9
*Pseudotsuga menziesii*	as *Picea*	0.9
Other conifers	as *Picea*	0.9
*Fagus* spp.	1.0	1.0
*Acer* spp.	as *Fagus*	0.9
*Alnus* spp.	as *Fagus*	0.9
*Betula* spp.	as *Fagus*	0.9
*Carpinus* spp.	as *Fagus*	1.0
*Fraxinus* spp.	0.8	0.8
*Populus nigra*	as *Fagus*	0.9
*Populus tremula*	as *Fagus*	1.0
*Quercus* spp.	as *Fagus*	1.0
*Robinia pseudoacacia*	as *Fagus*	0.9
*Salix* spp.	as *Fagus*	0.8
*Sorbus* spp.	as *Fagus*	0.9
*Tilia* spp.	as *Fagus*	0.8
*Ulmus*	as *Fagus*	1.0

Values of Badoux were modified from Gayer and Fabricius^28^.

All field measurements were recorded on paper copies of field record forms. The documents are available in the research collection of the WSL archive under “Wissenschaftliche Sammlung Ertragskunde” and partially also uncatalogued in the EFM archive. In 1974 data on measured stem dimensions from the field recording forms were converted to punchcards and over time they were also converted to a digital format. Branchwood data were processed in a separate project in 1984. This resulted in two independent datasets, one for stem dimensions (N = 38’864 individual tree data) and one for branch volume (N = 14’712). These datasets were the basis for the existing volume models in the Swiss NFI^16,19^. Documentation of this work is available on handwritten notes, and for the branchwood data in 1984 also in a detailed project proposal. Due to missing metadata, it was not straightforward to recognize whether a correlation between the two datasets existed. The here presented dataset (henceforth current dataset^29^) is the result of research on the provenance of the initial separate stem and branch datasets that allowed to link the measured data for individual trees. Furthermore, to extend the DBH and elevation range as well as to increase the sample size of trees from uneven-aged forests, the current dataset was expanded by digitizing additional tree data from the original paper copies.

## Data Records

**Fig. 4:**
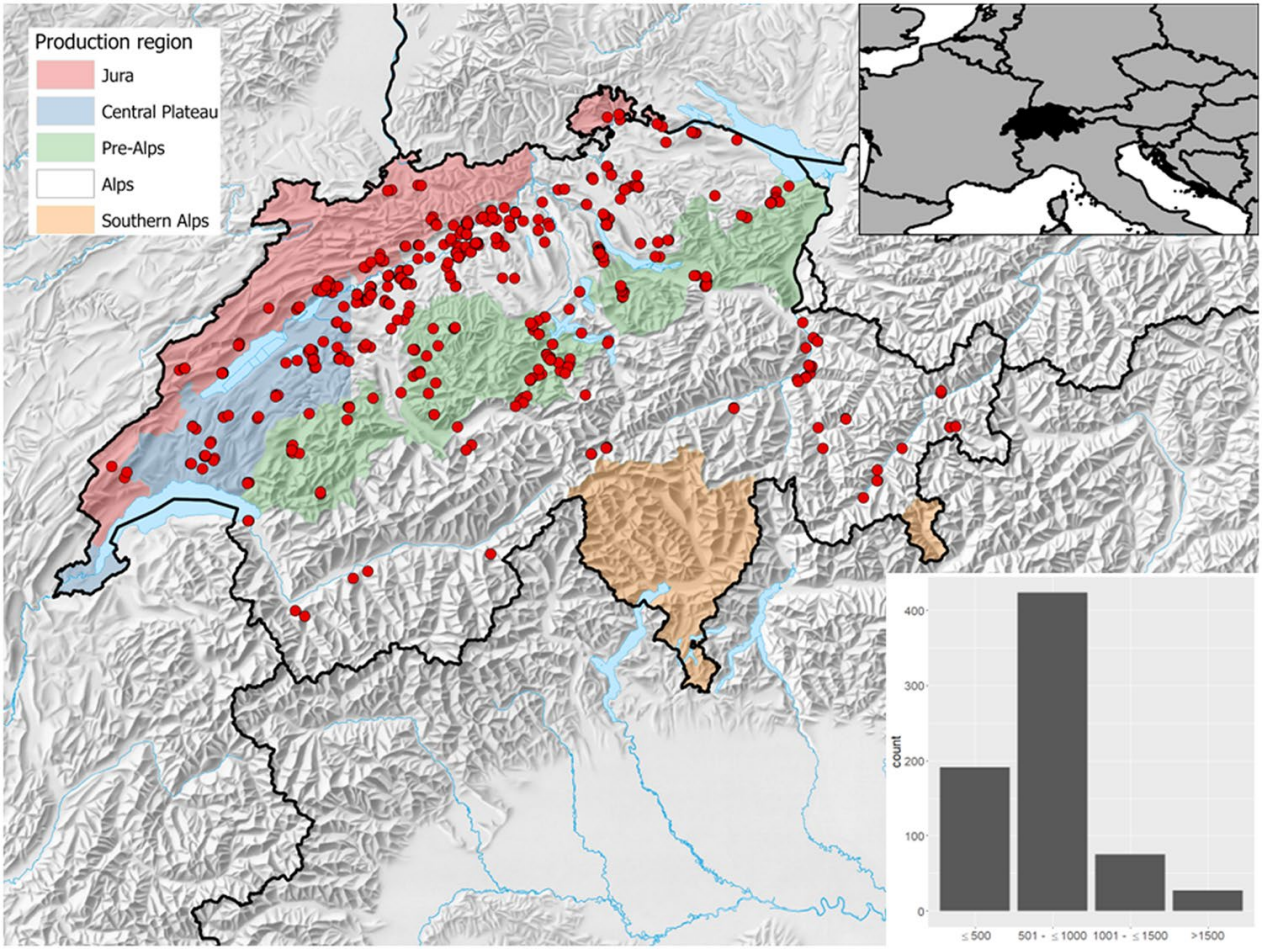
Spatial distribution of the 716 EFM plots with stem and branch data. Note that sites may overlap and are not visible and that for 52 plots no detailed spatial information was available. The five production regions represent a classification used in the Swiss National Forest Inventory indicating relatively homogeneous growth and wood production conditions (Glossary in Fischer and Traub^15^). The insert presents the elevation distribution of the plots by 500 m classes.

**Fig. 5:**
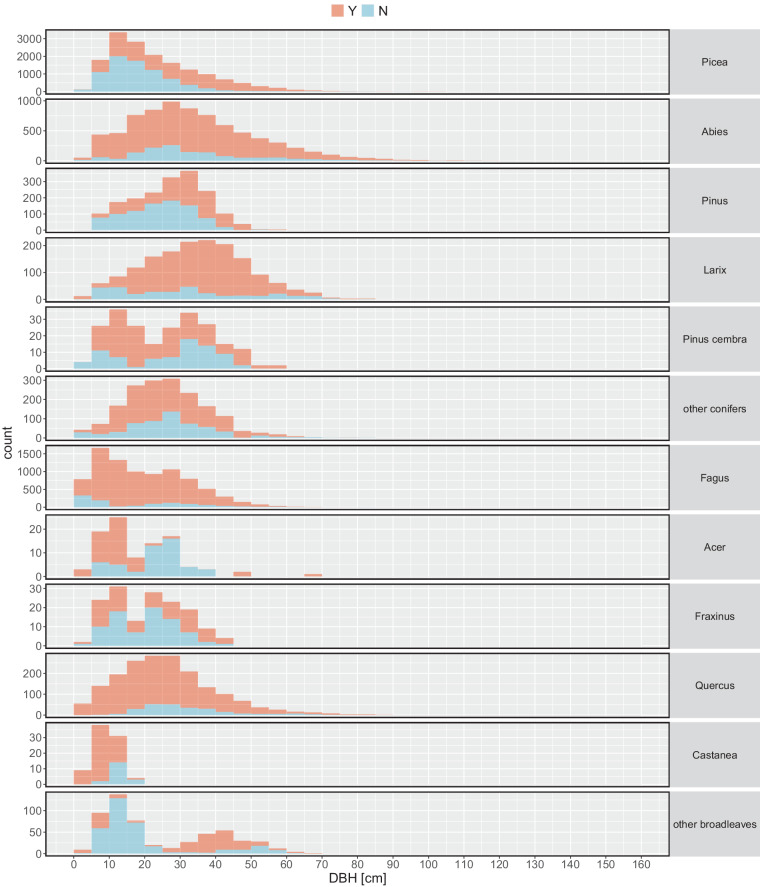
DBH distribution in 5 cm bins of trees in the current dataset by main tree species (NFI classification, cf. Table 1) with corresponding branch volume (coarse and/or fine, i.e. diameter threshold of 7 cm) data (‘Y’) or with stem measurements only (‘N’). Note that 24 observations with DBH > 1000 mm are not shown.

**Fig. 6:**
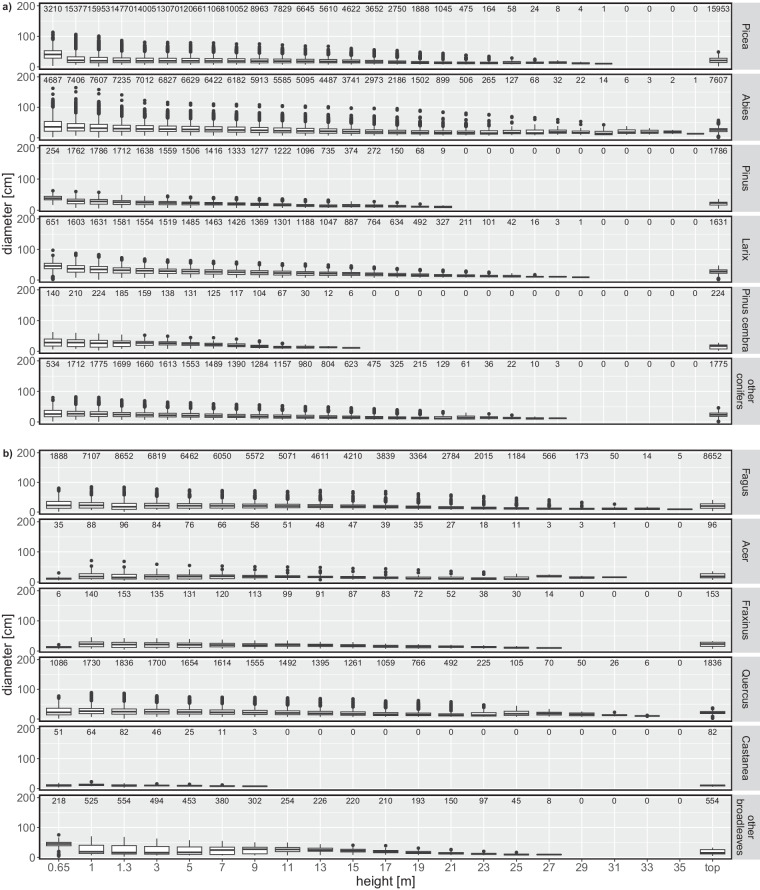
Boxplots of the diameter of stem sections at 0.65 m, 1.3 m (i.e., DBH) and starting at 1 m every 2 m until the lower threshold of 7 cm is reached, as well as the diameter at half the length of the tree top (i.e, the part of the stem where it has a diameter of 7 cm and the full height) by main tree species (NFI classification, cf. Table 1) for (**a**) conifers and (**b**) broadleaves. The values on top of each boxplot give the sample size. Note the different x scale rand for tree height between (**a**) conifers and (**b**) broadleaves.

**Fig. 7:**
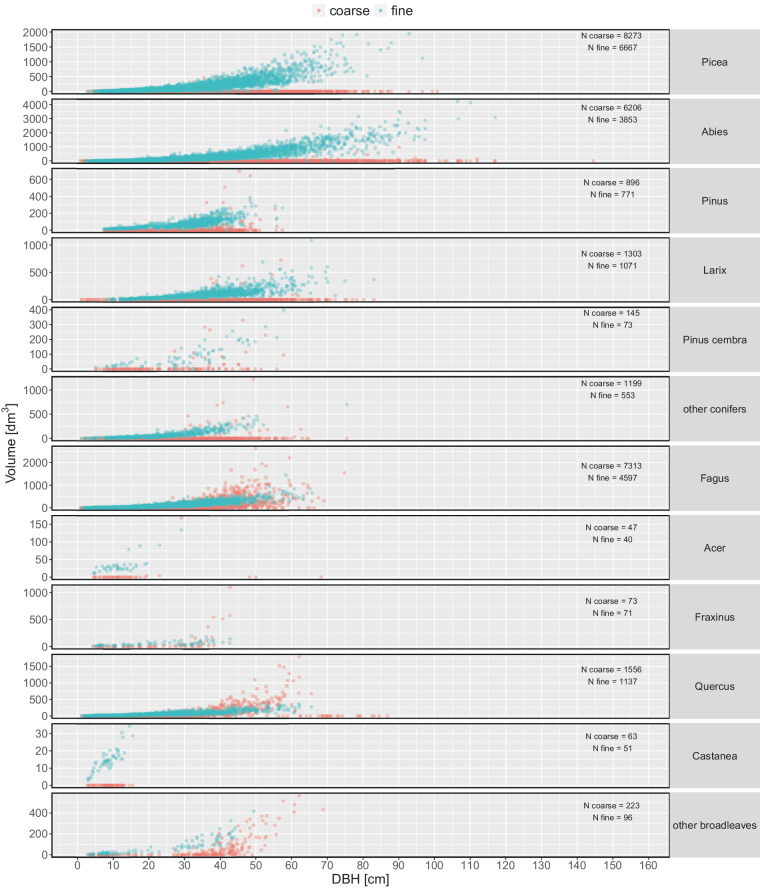
Volume of coarse branchwood and total of fine woody elements < 7 cm in diameter, i.e. including tree top, by main tree species (NFI classification, cf. Table 1). The point transparency indicates point density. Sample sizes are given on the right of each panel. Note the different y axis range for each species.

The current dataset^29^ contains measurements of 40’349 individual trees collected on 768 EFM sample plots (Fig. 4, Table 4). All available variables including their units and summary statistics are presented in Table 4. Figure 5 shows the DBH distribution, Fig. 6 the diameter distributions of different stem sections, and Fig. 7 the calculated volume of coarse and fine branches in relation to tree DBH. The dataset covers information from 768 plots (Fig. 4, Table 4), excluding subplots, collected at variable intervals in the period 1888 to 1974. It includes latitude, longitude and elevation of the plot centre. Site identifiers for each record can be used to derive further site metadata^18^. The tree data include information on.

- tree species (N = 28),
- tree age (based on year ring count; mean 73 years),
- DBH (mean 255 mm; Fig. 5),
- total length of the stem (i.e. tree height; mean = 219 dm),
- length of the coarse stemwood (from the base of a tree to the diameter threshold of 7 cm (mean 180 dm),
- length of the final section of the coarse stemwood (mean 7 dm),
- length of the tree top (i.e. starting where the stem diameter is 7 cm to the end of the stem; mean = 39 dm),
- mean of crosswise measured diameters over bark along the stem at 0.65 m and every 2 m starting at 1 m from the tree base up to the length of the coarse stemwood (Fig. 6),
- diameter of the final section if less than 2 m at half its length (mean 48 mm),
- diameter of the tree top (part of the stem where D < 7 cm measured at half its length (mean 34 mm),
- volume of coarse branches (derived from the measured diameters at the middle of one-meter sections(mean 16 dm^3^; Fig. 7),
- volume of all fine woody parts (ie. fine branches and tree tops derived from the measured weight of standardized bundles (mean 144 dm^3^; Fig. 7), and
- volume of fine branches (where measured separately; derived from the measured weight of standardized bundles mean 141 dm^3^).

**Table 4:** Site (total N = 768) excluding subplots data.

Variable name	Definition	Value range	N
SiteID	Site descriptor; 8‐digit code^17^ where the first five digits identify the main site, the final three digits subplots.	01001000–62007004	1028
Lat*	Latitude of the plot centre [degrees north]	46.08°–50.57°	716
Long*	Longitude of the plot centre [degrees west]	6.15°–10.24°	716
Elev*	Elevation [meter above sea level] derived from a digital elevation model	310–2000	716
NFI_PR	NFI Production region^15^	Jura, Plateau, Pre-Alps, Alps	768
InvYear	Inventory year	1888 - 1974	40’349
StandAge	Age structure	- even-aged	33’044
		- uneven-aged	6’727
StandComp	Tree species composition	- pure	23’685
		- conifer mixed (>75% conifers)	8’758
		- broadleaved mixed (>75% broadleaves)	620
		- conifer-broadleaved mixed	5’490

Note that 52 sites were abandoned after, e.g. clearcutting and have no detailed location information (Lat, Long, Elevation).

The quality controlled (see section ‘Technical Validation’) data^29^ are stored in table format as comma-separated file (.csv). The file is available from the environmental data portal EnviDat of the Swiss Federal Institute for Forest, Snow and Landscape Research WSL (10.16904/envidat.486). Missing values or not measured variables are denoted by NA. Values of ‘0’ indicate true values, e.g. in the case of coarse branchwood on spruce (*P*. *abies*) trees that generally only possess fine branches^30^.

## Technical Validation

**Fig. 8:**
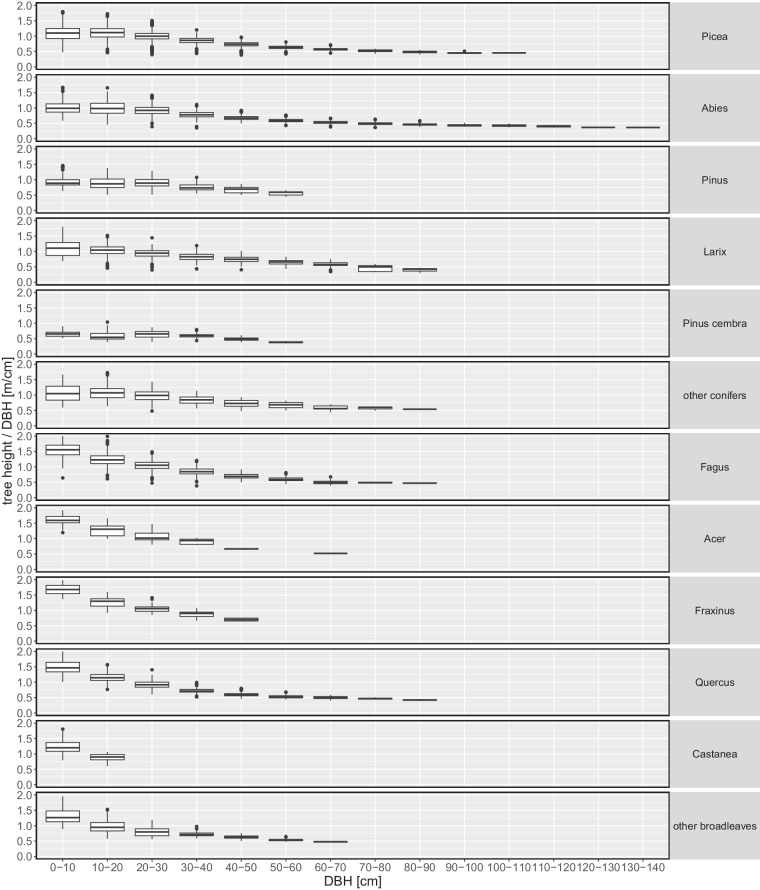
Slenderness ratio (total tree height/DBH) per 10 cm DBH bins by main tree species (NFI classification, cf. Table 1).

In a first step, the two initially available and separate digital datasets were assessed for consistency with field records and plausibility. The examination of the field recording forms also allowed the tree measurement procedure to be confirmed. Detailed information on the field procedure with cross-reference to field recording forms are available in section 3.2 in Didion, *et al*.^31^. The plausibility of tree attribute values was evaluated using consistency checks to identify, for example, duplicate tree records, cases where the diameter of stem sections decreased from the base to the top of the stem, or where tree height was less than the length of the merchantable part of the stem. Outlier detection was used to examine values of individual variables and in combination, for example the height to DBH ratio (Fig. 8), and diameters along the stem. The quality control and merge of the stem and branch datasets was achieved in several successive steps making use of the common variables, i.e. site information, inventory year, tree species, DBH, diameter at 7 m, and total height. The correct merge by individual trees was verified by comparing with field recording forms. Duplicates were removed and records that appeared not plausible were verified based on manual entries in field recording forms and corrected or otherwise left unchanged. The current dataset thus consists of verified and complete tree records.

## Usage Notes

Although this dataset with consistent and detailed measurements of nearly 40’000 individual trees is very comprehensive, it should be noted that:

- particularly the Swiss regions of the Southern and Western Alps are not well represented with only few sites in the Valais and none in Ticino (Fig. 1);
- mountain forest at higher elevations (>1500 m) are poorly covered in comparison to the forest distribution based on the Swiss NFI;
- the majority of the data comes from homogenous, even-aged forests.

The dataset provides an empirically derived stem and branch volume. It can be used to calibrate allometric functions with variables that are easy to measure in the field such as DBH, tree height as well as a second diameter. The comprehensive dataset can also be used to examine alternative stem volume estimations based on, for example, a cylindric first section and adding truncated cones using the mid-diameters of further sections to represent top and bottom, or taper functions^11,32^.

## Data Availability

All data processing including quality controls and figure generation was done using the language and environment for statistical computing R version 4.2.1^33^ and the packages data.table^34^, ggplot2^35^, and dplyr^36^.
